# Quantitative Analysis of Annual Training Volume and Periodization Patterns in Elite Female Cross-Country Skiers Using GPS Monitoring: A Three-Athlete Case Study

**DOI:** 10.3390/bioengineering13040429

**Published:** 2026-04-07

**Authors:** Xiangzi Xiao, Soyoun Moon, Yonghwan Kim, Yongchul Choi

**Affiliations:** 1 Department of Education, Taylor’s University, Jalan Taylor’s, Subang Jaya 47500, Malaysia; xiaoxiangzi@sd.taylors.edu.my; 2 Department of Physical Education, Gangneung-Wonju National University, Gangneung 25457, Republic of Korea; soabc2309@naver.com; 3 Laboratory of Integrative Physiology, Department of Health and Human Performance, University of Houston, Houston, TX 77204, USA

**Keywords:** cross-country skiing, training intensity distribution, five-zone model, periodization, female athletes, polarized training, case series

## Abstract

**Background**: The Global Positioning System (GPS) and wearable monitoring technologies are increasingly applied in sport science to quantify training load; however, data from female cross-country skiers in nations with emerging competitive programs remain scarce. This case series covering the complete national team roster analyzed the complete annual training cycle of the Korean women’s national cross-country skiing team (KCF) using GPS and heart rate-based wearable sensors. **Methods**: All three national team members were monitored throughout the 2022–2023 season (52 weeks), structured into General Preparation Period 1 (April–July), General Preparation Period 2 (August–November), and Competition Period (December–March). Individualized five-zone intensity thresholds were established through graded exercise testing on a roller ski treadmill with ventilatory threshold and blood lactate determination, independently assessed by two exercise physiologists (PhD level). **Results**: The total annual training volume was 667.72 h, comprising roller/on-snow skiing (54.0%), running (23.3%), and strength training (22.7%). The endurance-only intensity distribution demonstrated a polarized pattern (Zones 1–2: 91.5%). The total annual training distance reached 4673.30 km. The mean FIS points were 108.46 ± 38.60, and the mean VO_2_max was 60.17 ± 6.11 mL·kg^−1^·min^−1^. **Conclusions**: When benchmarked against world-class female (WCF) standards (800–950 h annually), the overall training volume was approximately 18–30% lower. The relative strength training allocation (22.7%) exceeded typical WCF values (10–15%). These observations should be interpreted cautiously given the small sample size and cross-study comparison design, using published literature-based benchmarks.

## 1. Introduction

Cross-country skiing demands exceptional aerobic capacity, muscular endurance, and technical proficiency [1,2]. Elite cross-country skiers demonstrate the highest VO_2_max values among endurance athletes, with male skiers exceeding 80 mL·kg^−1^·min^−1^ and female skiers reaching approximately 70 mL·kg^−1^·min^−1^ [1,3,4]. These capacities are developed through systematic, high-volume training programs [5].

Solli et al. [6] provided a landmark analysis of the world’s most successful female cross-country skier, documenting approximately 90% of annual endurance training at low intensity, with high-intensity training concentrated during competition season. This polarized approach has been identified as a distinguishing feature of Norwegian programs [6,7,8].

Training periodization is fundamental to optimizing long-term athlete development [9,10]. World-class cross-country skiers structure the annual cycle into preparation and competition phases [10,11]. Block periodization of HIT is particularly effective [12], and both block and traditional periodization can lead to world-class performance [13].

Despite this, the majority of research has focused on male athletes from traditional Nordic nations [14,15]. National-level investigations have concentrated on male cohorts [16,17,18,19]. Female-specific research remains limited [8,13,20]. Female athletes exhibit distinct physiological characteristics including differences in substrate utilization [21], hormonal fluctuations [22], and potentially different recovery kinetics [23]. Strength training is typically 10–15% in WCF programs [15,24]. These sex-specific factors are directly relevant to the present study, as our analysis of KCF athletes’ training load and intensity distribution may reveal patterns that differ from male-dominated literature, thereby informing evidence-based recommendations for female cross-country skiers.

At the 2026 Milano–Cortina Winter Olympic Games, the women’s program included a 50 km mass start event for the first time [25]. This equalization of race distances places greater demands on endurance capacity in female athletes.

Critically, nearly all published training analyses originate from Scandinavian or Central European nations, where the sport benefits from extensive snow seasons (6–7 months), well-established coaching traditions, and large athlete pools [8,10]. Data from athletes in East Asian or other non-traditional skiing nations remain virtually absent from the literature.

Despite these practical imperatives, cross-country skiing research in Korea has primarily focused on male athletes. This study utilized GPS technology and wearable heart rate monitors to objectively quantify the annual training load of three women’s national cross-country ski team athletes across a full competition season [26].

Specifically, this study addressed the following research questions: (1) What is the annual training volume and its distribution across periodization phases? (2) How does training intensity distribute across five heart rate zones? (3) How do these parameters compare with published world-class female standards?

We hypothesized that KCF athletes would exhibit lower total training volumes, a relatively higher proportion of strength training, and lower absolute hours of high-intensity endurance training compared with published WCF benchmarks.

## 2. Methods

### 2.1. Study Design and Participants

This study employed a prospective, observational, longitudinal design over a complete annual training cycle (52 weeks). The study protocol was approved by the IRB prior to data collection (GWNUIRB 2021-11, 25 February 2021), and monitoring was conducted prospectively throughout the 2022–2023 training season. All three members of the KCF participated. Because the sample encompasses the entire national team roster, this constitutes a case series including the complete national team roster [27]. All participants provided written informed consent. The study was conducted in accordance with the Declaration of Helsinki. To protect participant anonymity, data are presented as group means ± SDs. The participant characteristics are shown in Table 1.

### 2.2. Periodization Structure

The annual training cycle was divided into three phases [10,14]: General Preparation Period 1 (GPP1: April–July, 17 weeks), General Preparation Period 2 (GPP2: August–November, 17 weeks), and Competition Period (CP: December–March, 18 weeks). Training was categorized into three modalities: (1) roller skiing and on-snow skiing, (2) running, and (3) strength training.

### 2.3. Training Intensity Zone Determination

**Figure 1:**
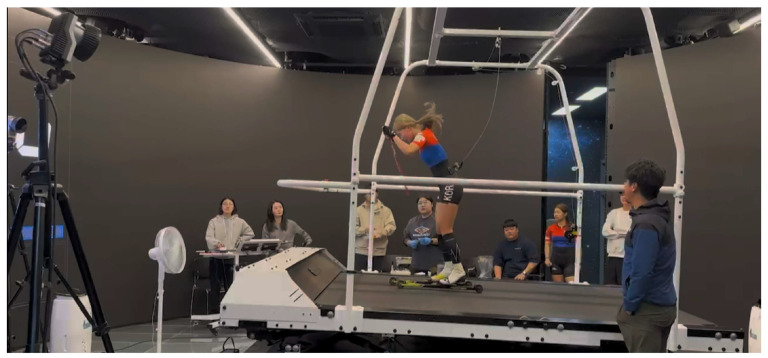
Graded exercise test (GXT) on a roller ski treadmill with the COSMED K5 wearable metabolic system for determination of HRmax, VT1, and VT2.

Individual training intensity zones were established using a five-zone heart rate model based on previous studies [6,7]. Each participant underwent a GXT on a roller ski treadmill (Figure 1). Respiratory gas exchange was continuously measured using the COSMED K5 (C09090-01-99, COSMED, Rome, Italy), and capillary blood samples were collected at the end of each stage for blood lactate analysis.

VT1 was determined as the first non-linear increase in VE/VO_2_ without a concurrent increase in VE/VCO_2_, combined with the V-slope method and the first lactate inflection point. VT2 was identified as simultaneous increases in both VE/VO_2_ and VE/VCO_2_, with RER above 1.0 and the onset of blood lactate accumulation (OBLA). All thresholds were independently assessed by two exercise physiologists (PhD level), and resolved by consensus.

Zones 1–2 were below VT1, Zone 3 was between VT1 and VT2, and Zones 4–5 were above VT2 [7]. Zones 1–2 were combined as LIT and Zones 4–5 as HIT, consistent with endurance training conventions [7,27].

### 2.4. Training Monitoring and Data Collection

Training data were collected using the Catapult Vector S7 (ICC 0.97–1.00 [28], Catapult Innovations, Melbourne, VIC, Australia). These reliability data were from court-based sports; GPS accuracy may be reduced in forested/mountainous terrain. Records were cross-checked with coaching staff. Heart rate was recorded at 1 Hz via Polar H10 (Polar Company, Kempele, Finland) [29]. Raw data were filtered using a 5-beat moving average; points outside ±20% of HRmax were excluded. HR and GPS data were time-synchronized.

### 2.5. Strength Training Characterization

Strength sessions were classified by objective: maximal strength (3–5 × 3–6 reps at 80–90% 1RM), muscular power (3–4 × 4–6 reps), muscular endurance (2–3 × 15–25 reps at 50–65% 1RM), and core/stability. GPP1: 3–4/week; GPP2: 2–3/week; CP: 1–2/week.

### 2.6. Comparative Data Sources

WCF benchmarks prioritized female-specific data: Solli et al. [8] (~940 h/yr, ~91% LIT), Sandbakk et al. [20], Walther et al. [30], and Osborne et al. [27] for contemporary Norwegian female data. The general references [1,5] were confirmed applicable by [8,30]. 

### 2.7. Data Analysis

Given the case-series design (n = 3), inferential statistics were not employed [31]. Data are presented as group means ± SDs. The intensity distribution is reported using two denominators: %total and %endurance-only. This approach follows recommendations for case-series research [32].

## 3. Results

### 3.1. Annual Training Volume by Periodization Phase

**Figure 2:**
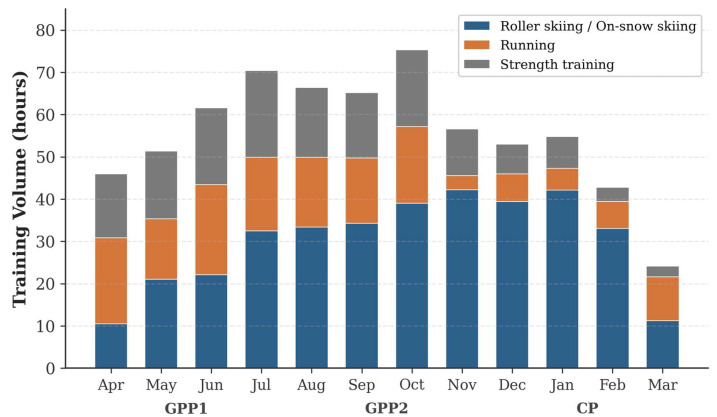
Monthly distribution of training volume (hours) by modality. Blue: roller skiing/on-snow skiing; orange: running; gray: strength training.

The total annual training volume was 667.72 h: roller skiing/on-snow skiing, 360.79 h (54.0%); running, 155.29 h (23.3%); and strength training, 151.64 h (22.7%). During GPP1, the volume progressively increased from 45.98 h to 70.45 h. GPP2 maintained high monthly volumes (56.62–75.34 h), with a peak in October (75.34 h). During CP, the volume decreased from 52.98 h (December) to 24.14 h (March) (Table 2 and Figure 2).

### 3.2. Annual Training Distance

The training distance distribution is presented in Table 3. The total annual distance was 4673.30 km: roller skiing/on-snow skiing, 3783.20 km (80.9%), and running, 890.10 km (19.1%). The peak monthly distance occurred in October (491.68 km). During the CP, on-snow skiing dominated (>90% of distance), with March recording the lowest distance (126.82 km).

### 3.3. Annual Training Intensity Distribution

**Figure 3:**
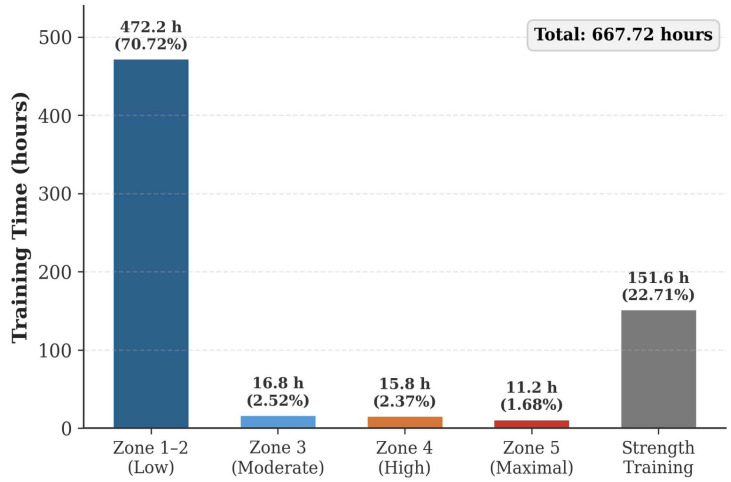
Annual training intensity distribution (hours). Zones 1–2: low-intensity; Zone 3: moderate-intensity; Zone 4: high-intensity; Zone 5: maximal-intensity; Strength: resistance training.

The annual intensity distribution (Table 4, Figure 3) showed that Zones 1–2 comprised 472.22 h (70.72% of total). When expressed as endurance-only (516.08 h), Zones 1–2 accounted for 91.5%, and Zones 3–5 for 8.5%. The distinction between these two denominators is noted for clarity in subsequent comparisons.

### 3.4. Phase-Specific Training Intensity Distribution

During GPP1, Zones 1–2 averaged 37.04 h/month, with Zones 4–5 averaging 1.35 h/month. During GPP2, Zones 1–2 increased to 46.89 h/month, and Zones 4–5 averaged 2.03 h/month. During CP, Zones 1–2 averaged 34.12 h/month, and Zones 4–5 increased to 3.38 h/month (+67% from GPP2) (Table 5).

### 3.5. Comparison with WCF Benchmarks

KCF athletes’ annual volume was approximately 18–30% below WCF norms. The strength training proportion was approximately double that reported for WCF athletes, while absolute hours of low-intensity endurance training were correspondingly lower (Table 6).

## 4. Discussion

This study provides rare descriptive evidence from an emerging East Asian cross-country skiing program. With the exception of a few specific events, Korean winter sports generally have low international competitiveness. Cross-country, in particular, has a small pool of athletes, leading to research focused on men. Furthermore, this study utilized GPS and wearable devices to collect objective, quantitative data.

### 4.1. Training Volume Deficit

The total annual training volume (667.72 h) was approximately 18–30% below the WCF norms of 800–950 h [8,15]. It should be noted that these WCF benchmarks are derived from published studies on different athlete cohorts, assessed during different time periods, and using potentially different training categorization methods. As such, they serve as approximate reference points rather than direct comparisons. Solli et al. [8] documented approximately 940 h/year for the world’s most successful female cross-country skier. Osborne et al. [27] more recently reported annual training volumes of 682–870 h for Norwegian female cross-country skiers across different competitive levels, providing additional contemporary context for interpreting the KCF data. Critically, the volume deficit was concentrated in endurance-specific training (516.08 h vs. an estimated 700–850 h in WCF athletes), while the strength training volume (151.64 h) was comparable or higher in absolute terms. This pattern suggests that the training program prioritizes strength development at the expense of the high-volume, low-intensity endurance training that underpins aerobic adaptations such as mitochondrial biogenesis, capillary proliferation, and enhanced fat oxidation capacity [7,33]. These observations suggest that progressive increases in total endurance training volume, particularly in Zones 1–2, may represent the most impactful modification for KCF athletes, though this inference requires prospective validation.

### 4.2. Strength Training Allocation: Strategic Choice or Structural Imbalance?

The strength training proportion (22.7%) substantially exceeded the 10–15% typically reported for WCF skiers [24,34]. This observation warrants nuanced interpretation. Korean female athletes may face specific physical challenges—including relatively lower upper body mass and strength compared with Nordic counterparts—that coaches address through increased strength training emphasis. Additionally, the Korean coaching tradition has historically prioritized strength development as a means of compensating for limited on-snow training opportunities due to climatic and infrastructural constraints.

The Korean training context differs fundamentally from that of traditional Nordic skiing nations in several respects. First, the natural snow season in Korea is limited to approximately 5 months (November–March), compared with 6–7 months in Scandinavian countries, substantially reducing opportunities for on-snow sport-specific training. Second, cross-country skiing training facilities in Korea are fewer and less specialized than those available in Nordic countries, with limited access to prepared ski trails for roller ski training during the off-season. Third, the athlete pool is considerably smaller, meaning fewer training partners for group-based high-intensity sessions that characterize Nordic programs. Fourth, Korean athletes have less frequent exposure to international competition, limiting opportunities for race-specific conditioning and tactical development. These contextual factors may partly explain both the lower total training volume and the emphasis on strength training as a controllable training modality.

However, the disproportionate allocation carries a clear trade-off: reduced time available for sport-specific endurance training. World-class female skiers maintain strength training at approximately 10–12% of total volume while achieving superior endurance adaptations [8,34]. Rather than simply reducing strength training volume, a more effective approach may involve improving the efficiency and specificity of strength sessions (e.g., transitioning from general hypertrophy to sport-specific power and strength-endurance) while gradually reallocating the freed time toward Zone 1–2 endurance work.

### 4.3. Training Intensity Distribution: Clarifying the Denominator

A key finding of this study is that the apparent training intensity distribution depends critically on whether strength training is included in the denominator. When expressed as a proportion of total training time, Zones 1–2 represented 70.72%—well below the 80–90% benchmark [7,8]. When expressed as a proportion of endurance-only time, Zones 1–2 represented 91.5%, which superficially aligns with the polarized TID model. However, this alignment is misleading: the absolute hours of Zone 1–2 endurance training (472.22 h) remain substantially below the approximately 700–800 h accumulated by WCF athletes [5,8]. Thus, the fundamental issue is not the relative intensity distribution within endurance training, but rather the insufficient total endurance training volume, caused in part by the excessive strength training allocation. To avoid ambiguity, we recommend that future studies on cross-country skiing TID report intensity distributions using both denominators (total training time and endurance-only time) and clearly state which is being used in each comparison.

### 4.4. Periodization and Phase-Specific Intensity Modulation

Phase-specific analysis (Table 5) revealed that high-intensity training (Zone 4–5) increased from GPP2 (2.03 h/month) to CP (3.38 h/month), a relative increase of 67%. While this directional change is consistent with the periodization principle of increasing intensity during the competition phase [13,14], the absolute magnitude of CP high-intensity training remains modest compared with WCF athletes, who strategically concentrate HIT blocks during the competition season [8,13]. Solli et al. [8] documented that the world’s best female skier substantially increased her HIT volume during the competition season while reducing her total training volume—a pattern of “intensification with tapering” that was not clearly evident in KCF athletes.

These observations suggest that practical implementation could consider 2–3 week HIT blocks during the pre-competition and in-competition phases, using either block periodization [35] or mixed approaches [13], while maintaining a predominantly low-intensity training base. The principle of avoiding the “grey zone” of moderate-intensity training (Zone 3) in favor of polarized training between clearly low and clearly high intensities has been advocated in the literature [7].

### 4.5. Sex-Specific Considerations and Health Implications

As this study did not collect menstrual cycle or energy availability data, detailed discussion of sex-specific physiological responses is limited. However, it should be noted that menstrual cycle phase can influence substrate utilization, thermoregulation, and perceived exertion [22], potentially affecting the quality of and recovery from high-intensity training sessions.

Furthermore, the high relative energy expenditure associated with endurance training in female athletes raises concerns about relative energy deficiency in sport (RED-S), including menstrual dysfunction, impaired bone health, and compromised immune function [31]. Any recommendations to increase total training volume must therefore be accompanied by appropriate nutritional support and regular health monitoring. The relatively high strength training allocation observed in KCF athletes may, in this context, offer a protective benefit for bone mineral density—a consideration that should be weighed against the aerobic training volume trade-off.

### 4.6. Implications in Practical Training

Based on the patterns observed in this case series, the following considerations may warrant attention in future training planning, though they should be interpreted cautiously given the study’s observational design: (a) these observations suggest that progressive increases in total endurance training volume (targeting 750–850 h/year) with emphasis on Zones 1–2 could potentially narrow the gap with WCF benchmarks; (b) the optimization of strength training efficiency and gradual adjustment toward 12–15% of total volume; (c) strategic HIT block implementation during the competition period; (d) periodic GXT reassessment for accurate intensity zone calibration; and (e) the integration of health monitoring (including energy availability screening) into the training framework. The increasing endurance demands of the women’s cross-country skiing program—highlighted by the inclusion of the 50 km event at the 2026 Milano–Cortina Winter Olympics—underscore the importance of adequate endurance training volume for nations aiming to improve international competitiveness [25].

### 4.7. Limitations

Several limitations should be considered. First, while n = 3 represents the entire KCF (case series covering the complete national team roster), the small absolute number substantially constrains generalizability beyond this specific population. The training patterns described here reflect the practices of one national team during one competitive season, and may not be representative of Korean cross-country skiing more broadly, let alone other emerging skiing nations. Comparisons with WCF benchmarks derived from single-athlete case studies [8] should be interpreted as illustrative rather than definitive. Second, world-class comparisons are based on published literature from different cohorts, time periods, and potentially different intensity zone definitions; these are best interpreted as approximate benchmarks. Third, heart rate-based intensity monitoring has inherent limitations including cardiac drift during prolonged exercise, lag at exercise onset, and potential inaccuracy during intermittent high-intensity efforts. HR-based zone classification may not capture all dimensions of training stress, including neuromuscular load from strength training, glycogen depletion patterns, or the accumulated fatigue from altitude exposure during international travel. The VT-anchored zone calibration and periodic GXT reassessment partially mitigate these issues, but blood lactate-based validation was not performed during routine training sessions due to the practical constraints of continuous field monitoring. Fourth, competition performance indicators (FIS points trends, race times) were not longitudinally analyzed across the monitoring period, limiting the ability to directly link training characteristics to performance outcomes. Similarly, serial physiological assessments (e.g., VO_2_max measurements across the season) were not conducted, precluding the evaluation of training-induced fitness changes. Fifth, subjective training load data (e.g., session RPE) were not systematically collected. Future studies incorporating concurrent performance monitoring, blood lactate validation, and session RPE alongside heart rate-based tracking are warranted.

## 5. Conclusions

This case series, based on comprehensive GPS and wearable device monitoring of all three members of the Korean women’s national cross-country ski team during the 2022–2023 season, documents the following observations: (1) The total annual training volume (667.72 h) was approximately 18–30% below published WCF benchmarks, with the deficit primarily in endurance-specific training. (2) The relative endurance intensity distribution approximated a polarized model (91.5% Zones 1–2), but the absolute endurance volume (516.08 h) was substantially lower than WCF reference values. (3) The proportion of strength training (22.7%) was approximately double that typically reported for WCF athletes, potentially limiting the time available for endurance-specific work. (4) Competition-phase high-intensity training, though directionally appropriate in its periodization pattern, was modest in absolute magnitude. These descriptive findings, while limited by the small sample size and cross-study comparison design, suggest that strategic rebalancing toward a greater endurance volume may warrant consideration for nations with similar training profiles seeking to improve international competitiveness. Prospective intervention studies are needed to evaluate whether such modifications lead to measurable performance improvements.

## Figures and Tables

**Table 1:** Physical characteristics of the KCF participants (n = 3).

Variable	Mean ± SD	Note
Age (years)	24.0 ± 1.0	
Height (cm)	164.3 ± 3.2	
Body mass (kg)	51.0 ± 2.64	
Athletic career (yr)	14.0 ± 3.0	
National team (yr)	6.6 ± 3.5	
FIS points	108.46 ± 38.60	Lower = better
VO_2_max (mL·kg^−1^·min^−1^)	60.17 ± 6.11	GXT roller ski

**Table 2:** Monthly training volume (hours) across periodization phases.

Phase	Month	Skiing (h)	Running (h)	Strength (h)	Total (h)
GPP1	April	10.45	20.36	15.17	45.98
	May	21.01	14.30	16.09	51.40
	June	22.10	21.35	18.16	61.61
	July	32.50	17.39	20.56	70.45
GPP2	August	33.41	16.50	16.50	66.41
	September	34.25	15.48	15.48	65.21
	October	39.04	18.15	18.15	75.34
	November	42.24	3.33	11.05	56.62
CP	December	39.46	6.51	7.01	52.98
	January	42.11	5.13	7.58	54.82
	February	33.04	6.36	3.36	42.76
	March	11.18	10.43	2.53	24.14
Total		360.79	155.29	151.64	667.72

GPP1 (April–July); GPP2 (August–November); CP (December–March).

**Table 3:** Monthly training distance (km) across periodization phases.

Phase	Month	Skiing (km)	Running (km)	Total (km)
GPP1	April	145.04	131.43	276.47
	May	212.08	105.03	317.11
	June	237.78	146.10	383.88
	July	342.11	95.36	437.47
GPP2	August	372.51	87.40	459.91
	September	375.38	84.10	459.48
	October	373.28	118.40	491.68
	November	441.88	15.37	457.25
CP	December	428.73	27.83	456.57
	January	417.54	29.30	446.84
	February	322.44	37.36	359.80
	March	114.36	12.46	126.82
Total		3783.13	890.14	4673.28

GPP1, General Preparation Period 1 (April–July); GPP2, General Preparation Period 2 (August–November); CP, Competition Period (December–March).

**Table 4:** Annual training intensity distribution.

Zone 1–2 (h)	Zone 3 (h)	Zone 4 (h)	Zone 5 (h)	Strength (h)	Total (h)
472.22	16.83	15.79	11.24	151.64	667.72
70.72%	2.52%	2.37%	1.68%	22.71%	100%
(91.50%) *	(3.26%) *	(3.06%) *	(2.18%) *	—	516.08 *

* Endurance-only time (excluding strength).

**Table 5:** Phase-specific training intensity distribution (monthly averages, hours/month).

Phase	Zones 1–2 (h)	Zone 3 (h)	Zone 4 (h)	Zone 5 (h)	Strength (h)	Total (h)
GPP1	37.04	1.47	0.79	0.56	17.50	57.36
GPP2	46.89	1.68	1.19	0.84	15.30	65.90
CP	34.12	1.05	1.97	1.41	5.12	43.67

GPP1 (April–July, 4 month); GPP2 (August–November, 4 month); CP (December–March, 4 month).

**Table 6:** Comparison of training characteristics: KCF vs. WCF benchmarks.

Parameter	KCF (Present Study)	WCF
Annual training volume (h)	667.72	800–950 †
Endurance training volume (h)	516.08	~846 *
Zones 1–2 (% of total)	70.72%	~80% †
Zones 1–2 (% of endurance only)	91.5%	~91% *
Zones 3–5 (% of endurance only)	8.5%	~9% *
Strength (% total)	22.7%	~10–12% *
Annual distance (km)	4673	6000–8000 †

* Female-specific [8]; † Mixed-sex [1,5]; confirmed by [8,30].

## Data Availability

The data presented in this study are available on reasonable request from the corresponding author due to privacy.
